# The NOD1 and NOD2 in mandarinfish (*Siniperca chuatsi*): molecular characterization, tissue distribution, and expression analysis

**DOI:** 10.1186/s12863-018-0667-y

**Published:** 2018-08-17

**Authors:** Tiantian Gu, Lu Lu, Jingwen Wang, Lili Tian, Wenzhi Wei, Xinsheng Wu, Qi Xu, Guohong Chen

**Affiliations:** Key Laboratory of Animal Genetics & Breeding and Molecular Design of Jiangsu Province, Yangzhou University, Yangzhou, 225009 People’s Republic of China

**Keywords:** Mandarinfish, *NOD1*, *NOD2*, Gene expression, LPS, Poly (I:C)

## Abstract

**Background:**

NOD-like receptors (NLRs) are a family of cytoplasmic pattern recognition receptors (PRRs), of which *NOD1* and *NOD2*, are the main representative members. Many investigations have focused on the role of *NOD1* and *NOD2* in the innate immune response in Cypriniformes and Siluriformes. As an important economic fish in Perciformes, little is known about the function of *NOD1* and *NOD2* in mandarinfish (*Siniperca chuatsi*).

**Results:**

The full-length *NOD1* and *NOD2* cDNA sequence was obtained using reverse transcription polymerase chain reaction (RT-PCR) and rapid amplification of cDNA ends (RACE). The mandarinfish *NOD1* and *NOD2* cDNA sequences contain 3247 bp and 3257 bp, and encode 918 amino acids and 988 amino acids, respectively. Multiple sequence alignments showed that mNOD1 and mNOD2 share high similarity with that from other vertebrates. RT-PCR analysis revealed that relatively high levels of m*NOD1* and m*NOD2* mRNA were detected in gill and head kidney tissues, compared with the heart, spleen, liver, muscle, and intestine. In addition, the relative levels of m*NOD1* and m*NOD2* transcripts were significantly upregulated in three tissues when the fishes were challenged with LPS and Poly I:C, interestingly, the *NOD1* mRNA got peaked earlier than *NOD*2 after LPS induction in the spleen, gill, and head kidney, and during Poly I:C treatment, the NOD2 mRNA got peaked at 8 h in spleen and gill, while *NOD1* showed significant higher expression at 24 h post infection, besides, in head kidney, the NOD2 mRNA showed a great increasing trend and *NOD1* got peaked at 16 h. Therefore the m*NOD1* and m*NOD2* may act differently within different tissues in different time during antiviral and antibacterial defense.

**Conclusions:**

These results revealed the dynamic m*NOD1* and m*NOD2* expression during viral and bacterial infections, which suggested the *NOD1* and *NOD2* play important roles in innate immune of mandarinfish.

**Electronic supplementary material:**

The online version of this article (10.1186/s12863-018-0667-y) contains supplementary material, which is available to authorized users.

## Background

In vertebrates, the innate immune system is the fundamental defense mechanism, and plays a beneficial role in defending against invasion [1, 2]. The innate immune system rapidly recognizes conserved pathogen associated molecular patterns (PAMP) via the presence of the host’s own pattern recognition receptors (PRRs). At present, PRRs, which have been extensively studied, include Toll-like receptors (TLRs), retinoid acid–inducible gene-1 (RIG-I)-like receptors (RLRs), NOD-like receptors (NLRs), and DNA receptors. Studies have increasingly shown that RLRs and TLRs play an important role in the innate immunity of the organism and form the first line of defense against infectious pathogens [3–6]. Unlike TLRs and RLRs, NLRs are a recently identified large group of the intracellular PRR family characterized by multi-domain proteins, which contain an N-terminal protein-protein binding domain such as the caspase recruitment domain (CARD), pyrin (PYD), or baculovirus inhibitor of apoptosis repeat (BIR) domain, in addition to a central nucleotide oligomerization (NACHT) domain and a C-terminal leucine-rich repeat (LRR) domain [7–10].

However, the NLRs do not contain a signal peptide or transmembrane domain, which indicate their locations in the cytosol. The C-terminal LRR domain is the potential ligand recognition site and the NACHT domain mediates self-regulation and oligomerization. The N-terminus is responsible for protein–protein interaction, signal transduction and initiation of the downstream immune cascade [11, 12]. Based on the specific N-terminal domain present, NLRs are divided into three subfamilies including NODs (containing the CARD domain), NALPs (containing the PYR domain) and NAIPs (containing the BIR domain) [10, 13]. Both of NOD1 and NOD2 are part of NODs subfamily with CARD, NACHT and LRR, which share three functional domains in mammals [14]. Among them, NOD1 contains an N-terminal CARD domain, but NOD2 possesses two N- terminal CARD domain [15]. Previous studies have proved that NOD1 and NOD2 recognized bacterial and viral products through their C-terminal LRRs in mammals [16, 17]. Although the adaptive immune system of fish has well been developed, which can produce antibody response against infection. Innate immunity also plays an important role in protection against disease in fish. Some studies have found that after lipopolysaccharide (LPS) or polyriboinosinic polyribocytidylic acid (Poly (I:C)) stimulation, the expression of *NOD1* and *NOD2* was significantly upregulated in grouper fish [18], rainbow trout [19], carp [20], mrigal [21], catfish [22] and goldfish [23]. Similar results were also found in the spleen tissue of grass carp [24]. Upon ligand recognition, both NOD1 and NOD2 recruit RIPK to the receptors via CARD–CARD interactions, which lead to the activation of NF-kB and MAPK pathways [25]. The activation of NF-kB and MAPK mediated by NOD1 and NOD2 could induce transcription and production of inflammatory mediators and antimicrobial peptides, and induce apoptosis [15, 26]. Therefore, the NOD1 and NOD2 genes play an important role in the resistance of fish to the invasion of pathogenic microorganisms.

Among carnivorous freshwater fish, mandarinfish (*Siniperca chuatsi*), is a precious aquaculture species in China, with high economic value, but it is very sensitive to bacterial and viral infection. In recent years, outbreaks of disease epidemics such that of infectious spleen and kidney necrosis virus (ISKNV) [27, 28] and other epidemic diseases, such as Iridovirus [29, 30] and EHNV [31], have caused serious damage to the freshwater aquaculture industry in China; however, there have been few reports of the function of NOD1 and NOD2 genes against bacteria and virus in mandarinfishes. Based on this background, we performed the molecular cloning and characterization of the mandarinfish NOD1 and NOD2 genes and analyzed their expression levels during LPS and Poly (I:C) treatment in vivo. These data might facilitate a better understanding of the role of *NOD1* and *NOD2* in bacterial and viral infections, which is beneficial not only to the understanding of innate immunity mechanisms in mandarinfish, but also to monitor the disease occurrence in the aquatic culture industry.

## Methods

### Tissues

A total of fifty-seven mandarinfishes with 400-600 g in body weight were obtained from the the Gaoyou Dongshi Special Aquatic Products Company (Yangzhou, China). Fish were kept in aquarias with a flowthrough water system and aerated freshwater at 29 °C. Tissues, including heart, liver, spleen, gill, head kidney, muscle, and intestine, were collected from three healthy mandarinfishes. Tissue samples were harvested and immediately snap-frozen in liquid nitrogen and stored at − 80 °C until needed. Total RNA was extracted from all tissues with TRIZOL (Invitrogen, Carlsbad, CA, USA).

### In vivo challenge experiments

Fifty-four mandarinfishes were randomly divided into three groups, the eighteen fishes were injected with 500 μl Poly (I:C) (1 mg/mL) (Invivogen, CA, USA), the eighteen fishes were treated with 400 μl LPS (1 mg/mL) (Sigma, MO, USA), and the others were injected with saline, respectively. No fish treated with LPS or Poly (I:C) died At 0, 2, 4, 8, 16, 24 h after injection, spleen, gill, and head kidney tissues were collected from three fish. Tissue samples were harvested and immediately snap-frozen in liquid nitrogen and stored at − 80 °C until needed.

### Cloning of m*NOD1* and m*NOD2* cDNA

Total RNA was isolated from the spleen of healthy mandarinfish with TRIzol (Invitrogen, USA) according to the manufacturer’s instructions, and the quality of the isolated RNA was assessed by visualizing the ribosomal RNA bands after electrophoresis on a 1.0% agarose gel. The PrimeScript™ 1st Strand cDNA Synthesis Kit (TAKARA, Dalian, China) was used according to the manufacturer’s instructions with 1 μg of total RNA as a template to produce cDNA. Based on the conserved sequences of *Ictalurus punctatus*, *Larimichthys crocea*, *Lateolabrax japonicas*, and *Paralichthyidae*, forward and reverse primers for m*NOD1* and m*NOD2* were designed to obtain the internal region (Additional file 1: Table S1). For all genes, the polymerase chain reaction (PCR) amplification was conducted using LA Taq (TAKARA) with the following conditions: 94 °C for 5 min, 35 cycles of 94 °C for 30 s, 52 °C for 30 s, and 72 °C for 2 min, followed by one cycle of 72 °C for 10 min. Rapid amplification of cDNA ends (RACE)-ready first-strand cDNA was synthesized using a Takara RACE cDNA amplification kit (Takara, China) in accordance with the manufacturer’s protocol and RACE primers were designed according to the manufacturer’s protocol (Additional file 1: Table S1). Subsequently, RACE was performed on the 5′ and 3′ ends of the m*NOD1* and m*NOD2* cDNA. The sequences of m*NOD1* and m*NOD2* were submitted to GenBank under the accession numbers KY974318 and KY974317, respectively. All the primer sequences mentioned above are shown in Additional file 1: Table S1.

### Sequence alignment and homology analysis

A phylogenetic tree was constructed based on the deduced amino acid sequences using the Neighbour-Joining (NJ) algorithm within MEGA 6.0 program and a multiple sequence alignment was created by AlignIR V2.0. Clustal Omega (http://www.ebi.ac.uk/Tools/msa/clustalo/) was performed to construct multiple sequence alignments of the amino acid sequences of mNOD1 and mNOD2 proteins. The conserved domains of mNOD1 and mNOD2 were predicted using the NCBI Conserved Domains Database Tools (CDD Tools) (https://www.ncbi.nlm.nih.gov/Structure/cdd/).

### Expression of mNOD1 and mNOD2 mRNA in tissues

Total RNA was extracted from the tissues (heart, liver, spleen, gill, head kidney, muscle, and intestine) of healthy mandarinfish using TRIZOL (Invitrogen), 1 μg RNA was used with gDNase (TIANGEN, China) during reverse transcription for RT-PCR. The process included an initial phase at 42 °C for 3 min and then incubation at 42 °C 15 min followed by 95 °C for 3 min. The cDNA stored at − 80 °C. The primers are listed in Additional file 1: Table S1. The reaction conditions included 1 cycle at 95 °C for 5 min, followed by 35 cycles of 94 °C for 30 s, 60 °C for 30 s, and 72 °C for 30 s, and a final incubation at 72 °C for 10 min. The products of PCR were separated on 2% (*w*/*v*) agarose gels, and the β-actin gene was used as an internal standard for relative expression levels.

### Expression of mNOD1 and mNOD2 mRNA following poly (I:C) and LPS treatment in tissues

RNA extraction and cDNA synthesis were performed as described above. QuantStudio 5 (Applied Biosystems, Thermo Fisher Scientific, USA) was used to perform the assay, and qPCR was performed using SYBR Green Master Mix (Vazyme, Nanjing, China) to determine the pattern of mNOD1 and mNOD2 mRNA expression after challenge with Poly (I:C) and LPS, the following reaction conditions were conducted including 1 cycle at 95 °C for 5 min, followed by 30 cycles of 95 °C for 10 s and 60 °C for 30 s, and a final cycle of 95 °C for 15 s, 60 °C for 60 s, and 95 °C for 15 s. The relative expression of mRNA was calculated using the 2^-△△CT^ method, and the β-actin gene was used as an internal control.

### Statistical analysis

Data from all experiments are expressed by mean ± standard error. A database was established in Excel 2003 and the data were statistically analyzed with SPSS 13.0 using the one-way ANOVA Duncan method. *P*-values below 0.05 were considered statistically significant.

## Results

### cDNA cloning and sequence analysis of mandarinfish *NOD1* and *NOD2*

**Fig. 1:**
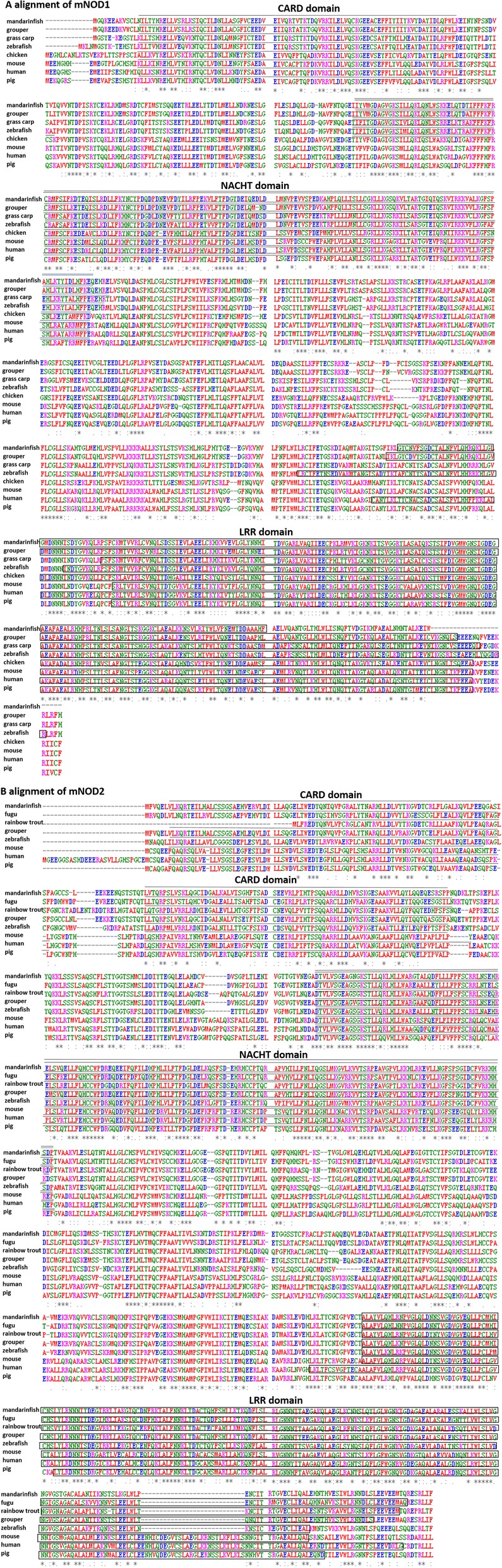
(**a**) Multiple alignments of NOD1 protein sequences from mouse (NM_172729.3), human (NM_006092.3), pig (NM_001114277.1), chicken (NM_001318438.1), zebrafish (XM_002665060.6), grouper (JX220894.1), grass carp (FJ937972.1), and mandarinfish (KY974318). (**b**) Multiple alignments of NOD2 protein sequences from mouse (NM_145857.2), human (XM_017023536.1), zebrafish (NM_001328044.1), pig (NM_001105295.1), fugu (NM_001042448.1), rainbow (NM_001201555.1), grouper (JX220895.1), and mandarinfish (KY974317). Abbreviations: CARD: caspase recruitment domain, NACHT: nucleotide binding/oligomerization domain, LRR: leucine-rich repeats Asterisks represent identical amino acids, and The symbols “.” or “:” denote conservative substitutions

Using RT-PCR and RACE technology, the full-length sequence of m*NOD1* was obtained, which included a 124-bp 5’ UTR, a 2757-bp open reading frame (ORF) and a 366-bp 3’ UTR, and encodes 918 amino acids. The m*NOD2* cDNA was cloned with 124-bp 5’ UTR, a 2967-bp ORF encoding 988 amino acids, and a 166-bp 3’ UTR. The primers used are shown in Additional file 1: Table S1. The sequences of m*NOD1* and m*NOD2* were submitted to GenBank (GenBank accession number KY974318 and KY974317, respectively). Furthermore, using the NCBI Conserved Domains Database Tools (CDD Tools) to predict the conserved domains of mNOD1 and mNOD2, we found that mNOD1 has one CARD domain (residues 13–97), one NACHT domain (residues 189–360), and seven LRRs (residues 670–868) (Fig. 1a), whereas mNOD2 includes two CARD domains (residues 7–91 and 115–195), one NACHT domain (residues 273–443), and six LRRs (residues 757–979) (Fig. 1b). A comparison of the coding sequence (CDS) and amino acid sequence of mNOD1 and mNOD2 to the NOD1 and mNOD2 genes of other species, including multiple alignments and amino acid sequence, are shown in Fig. 1a and b, respectively.

### Phylogenetic analysis

**Fig. 2:**
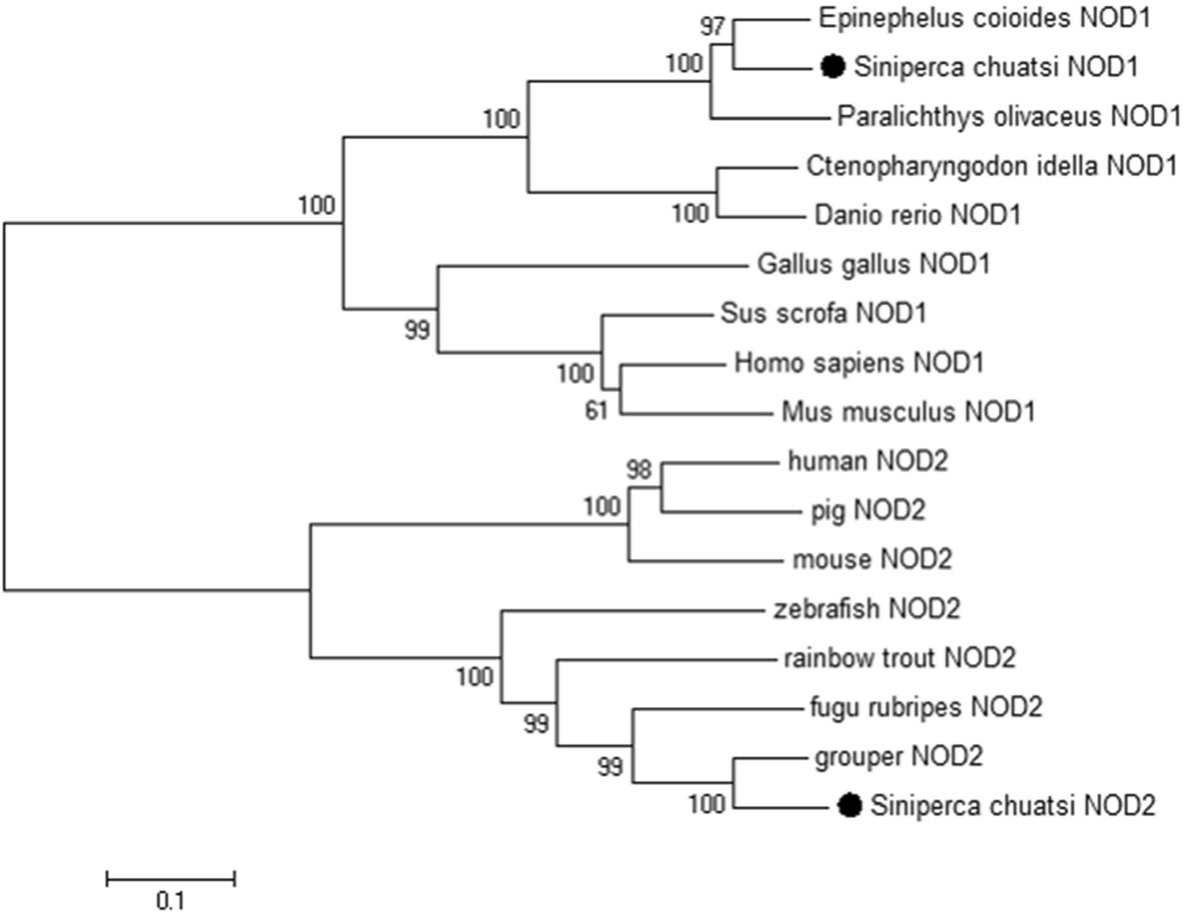
Phylogenetic analysis of avian, mammalian, and fish NOD1 and NOD2 was carried out. The tree was constructed by the neighbor-joining tree method using amino acid sequences aligned with MEGA6. The bar indicates the bootstrap value (%)

To evaluate the molecular evolutionary relationships between mNOD1 and mNOD2 and NODs orthologs in other vertebrates, a phylogenetic tree was constructed based on the amino acid sequences of mammalian, avian, and fish NODs. The inferred phylogeny of the NOD1 and NOD2 genes is presented in two distinct clusters (Fig. 2). Mandarinfish NOD1 was in the same branch as that of grouper, bastard halibut, grass carp, and zebrafish, but had a distant evolutionary relationship with mammals and birds, illustrating the closer relationships between mandarinfish NOD1 and those of other fish species. Mandarinfish NOD2 was most closely related to those of grouper, fugu, rainbow trout, and zebrafish, in a branch separate from that of the mammals.

### Tissue expression profiles of m*NOD1* and m*NOD2*

**Fig. 3:**
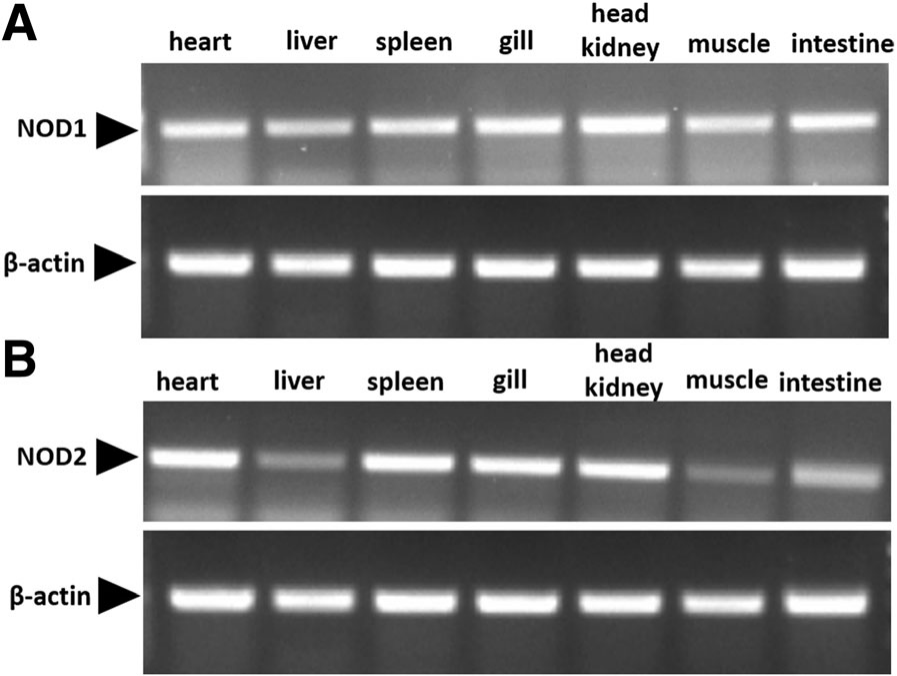
mRNA expression of m*NOD1* (**a**) and m*NOD2* (**b**) mRNA in various tissues. m*NOD1* and m*NOD2* were determined using RT-PCR and compared to the expression of β-actin. Tissues of three healthy fishes analyzed include heart, liver, spleen, gill, head kidney, muscle, and intestine

To determine the tissue expression levels of m*NOD1* and m*NOD2*, RT-PCR was performed. mRNA expression of m*NOD1* and m*NOD2* was detected in all seven tissues collected. We found that m*NOD1* and m*NOD2* mRNAs were highly expressed in heart, spleen, gill, and head kidney, particularly in head kidney and gill, and the expression of m*NOD2* was generally higher than that of m*NOD1* in these four tissues. On the other hand, relatively low expression of m*NOD1* and m*NOD2* was detected in the liver, muscle, and intestine (Fig. 3a and b).

### Expression of m*NOD1* and m*NOD2* in tissues after LPS and poly (I:C) treatment

**Fig. 4:**
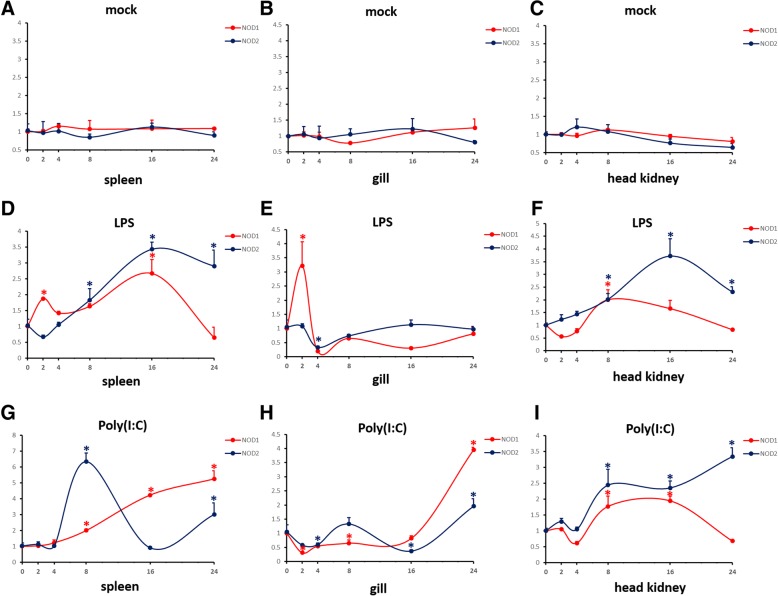
mRNA expression levels of m*NOD1* and m*NOD2* in vivo following saline, LPS or Poly (I:C) injection. (**a**, **b**, **c**) Fish were injected intraperitoneally with saline in the spleen, gill, and head kidney after 0, 2, 4, 8, 16, and 24 h post-injection, (**d**, **e**, **f**) LPS and (**g**, **h**, **i**) Poly (I:C). Gene expression was determined using real time RT-PCR and was normalized to β-actin. Relative transcription level was obtained by comparing the normalized expression levels between different time points. Vertical bars represent the mean ± S.E. (*n* = 3). Asterisks represent significant differences relative to controls (*P* < 0.05)

To further determine the regulation of m*NOD1* and m*NOD2* expression by LPS or Poly (I:C) in vivo, different tissues from treated and untreated groups were collected following treatment with saline、LPS or Poly (I:C). The mRNA expression levels of m*NOD1* and m*NOD2* in different tissues were detected by qRT-PCR at various time points after injection (0, 2, 4, 8, 16 and 24 h). The mRNA expression of mNOD1 and mNOD2 showed no significant change in different tissues when injecting saline. And the relative transcript levels of m*NOD2* in spleen and head kidney were upregulated at 16 h after LPS stimulation and then gradually decreased, and in gill m*NOD2* mRNA showed a gently trend; however, the peak time of m*NOD1* mRNA appeared 2 h after the infection in spleen and gill, and 8 h in head kidney, and in spleen, the m*NOD1* mRNA got peaked again at 16 h (Fig. 4d, e, f). However, following Poly (I:C) injection, the expression levels of m*NOD1* and m*NOD2* in these three tissues showed a persistently increased trend (Fig. 4g, h, i), with the exception of m*NOD2* in the spleen (Fig. 4g) and m*NOD1* in the head kidney (Fig. 4i).

## Discussion

Innate immunity is the first line of defense against infectious pathogens, and NLRs, which function as intracellular PRRs, play an important role in resistance to bacterial and viral infection [24, 32, 33]. In human [17], the deficiency of *NOD2* would cause Crohn’s disease and in grass carp [24], *NOD1* and *NOD2* could be upregulated by different immunostimulants. However, almost nothing is known about the homeostatic or pathogen-induced expression patterns of NLR transcripts in mandarinfish.

To better understand the mechanism of *NOD1* and *NOD2* function in mandarinfish against pathogenic microorganisms, we isolated and identified the NOD1 and NOD2 cDNA sequence and characterized their sequence. The conserved domains of mandarinfish predicted by the CCD tool (Fig. 1a and b) showed that both of them had similar conserved domains containing CARD-NACHT-LRR domains. NOD2 had one more CARD domain than NOD1, and NOD1 had seven LRRs, whereas NOD2 only had six; however, both of them had a similar structure to grouper and miiuy croaker [18, 34]. The high conservation of LRR domains in NOD1 and NOD2 implied that they may possess similar functional properties as those of other fishes in the recognition of pathogenic microorganisms and viruses [18]. Furthermore, amino acid homology analysis and phylogenetic analysis of mandarinfish NOD1 and NOD2 revealed that they shared high similarities with NOD1 and NOD2 proteins from other teleost fishes, but low similarities with those of mammals and birds. Our results showed that *NOD1* and *NOD2* were evolutionarily conserved not only in terms of their protein sequences but also in terms of functionally significant domains.

In healthy mandarinfish, the NOD1 and NOD2 genes were widely expressed in all the tissues collected. RT-PCR analysis revealed that m*NOD1* and m*NOD2* mRNA were expressed at higher levels in the heart, spleen, gill, and head kidney than in other tissues, particularly in the head kidney and gill; moreover, the expression of m*NOD2* was generally higher than that of m*NOD1* in these four tissues (Fig. 3a and b), although this result was markedly different from that observed in grouper [18], grass carp [24], mrigal [21] and channel catfish [35]. In grass carp, the highest expression of *NOD1* was in the liver, whereas *NOD2* was highly expressed in head kidney [24]. In mrigal, significant expression of *NOD1* was detected in the liver, whereas *NOD2* was abundantly expressed, with the highest levels observed in spleen [21]. As an important immune organ of fish, the head kidney has high expression levels of *NOD1* and *NOD2*, which is to be expected. In addition to the important immune organ, scattered lymphocyte germinal centers [35] are found in the mucous tissues of the gills, indicated that the gill might function as the first line of defense in the innate immune response, and high levels of NOD1 and NOD2 gene expression in the gill is also expected. Besides, the NOD1 and NOD2 genes of mandarinfish were also observed to be expressed at high levels in head kidney, speculating that *NOD1* and *NOD2* may take part in the anti-pathogen response.

NODs are a recently characterized group of pathogen recognition receptors, they are believed to be intracellular bacteria-recognizing receptor. The bacterial component, LPS, has been shown to influence NOD1 and NOD2 mRNA expression in mouse [36], rohu [37], and channel catfish [35]. In channel catfish, after bacterial induction, *NOD1* and *NOD2* were highly upregulated in head kidney and spleen [35]. A significant observation in this study was that the expression of NOD1 and NOD2 genes were upregulated in vivo following LPS stimulation, suggesting the involvement of these genes in antibacterial response. In addition, mandarinfish *NOD2* was activated to a greater extent, but *NOD1* peaked earlier than *NOD2* after LPS induction in spleen, gill and head kidney, indicating that *NOD1* and *NOD2* appear to play a unique role in initially sensing pathogenic and may work in different response time in defense against bacterial invasion.

In addition, some studies have showed that *NOD1* and *NOD2* could prove to participate in antiviral defense, such as human [38], olive flounder [39], rohu [20], grouper [18] and grass carp [24]. In human, *NOD1* was significantly induced following stimulation with human cytomegalovirus [38] and in grass carp activation of *NOD1* and *NOD2* was reported in reovirus infection [24]. In mandarinfish, both the NOD1 and NOD2 gene were found to be activated in viral infection, mimicked by treatment with the viral analogue Poly I:C, revealing that NODs are sensitive to the protective immune response against viral invasion. Interestingly, mandarinfish *NOD2* showed a more significant increasement when stimulated by PolyI:C than did *NOD1* in vivo, however, *NOD1* showed significantly higher expression at 24 h post infection in spleen and gill during PolyI:C stimulation, demonstrating that mandarinfish *NOD1* and *NOD2* may act differently within different tissues in antiviral defense. In addition, the expression levels of m*NOD1* and m*NOD2* mRNA after LPS stimulation generally peaked at an earlier time point than that observed with Poly (I:C) treatment, revealing that both of them were more sensitive to the antibacterial sensitive response than to viral invasion, indicating that these proteins more easily identify bacterial peptidoglycans and induce signaling pathways by acting as PRRs [40, 41], meanwhile, some other subfamilies of NOD-like receptors may be responsible for inducing *mNOD1* and *mNOD2* in response to LPS [22], which needs further studies.

## Conclusions

In summary, we first cloned and characterized the NOD1 and NOD2 cDNA sequences in mandarinfish, and demonstrated that the mandarinfish NOD1 and NOD2 genes are functionally similar to their counterparts from other teleosts. Transcriptional analysis showed ubiquitous expression of NOD1 and NOD2 gene in seven examined tissues. Expression analyses showed that NOD1 and NOD2 gene were significantly enhanced in vivo after LPS or Poly (I:C) stimulation, indicating that *NOD1* and *NOD2* may appear to play a unique role in initially sensing pathogenic and may work in different tissues in defense against bacterial and viral invasion. Taken together, our results revealed that the m*NOD1* and m*NOD2* might be involved in innate immune protection in mandarinfish.

## Additional file

Additional file 1: **Table S1.** Primers used in the study. (DOCX 14 kb)

## Abbreviations

BIR: Baculovirus inhibitor of apoptosis repeat
CARD: Caspase-activation and recruitment domain
CDD: Conserved Domains Database
CDS: Coding sequence
LPS: lipopolysaccharide
LRR: Leucine-rich repeat
NLRs: NOD-like receptors
ORF: Open reading frame
PAMP: Pathogen associated molecular patterns
PCR: Polymerase chain reaction
Poly (I:C): Polyriboinosinic polyribocytidylic acid
PRR: Pattern recognition receptors
RACE: Rapid amplification of cDNA ends
RLRs: RIG-1-like receptors
TLRs: Toll-like receptors
