# Supplementary material for: The NOD1 and NOD2 in mandarinfish (Siniperca chuatsi): molecular characterization, tissue distribution, and expression analysis
Source: BMC Genet. 2018 Aug 17;19:61. doi: 10.1186/s12863-018-0667-y (PMC6098665; doi:10.1186/s12863-018-0667-y)
Supplement: Supplementary file 1 — Table S1. Primers used in the study. (DOCX 14 kb) [file 12863_2018_667_MOESM1_ESM.docx]

**Table S1**

Primers used in the study.

| Primer name | Primer sequence (5'→3') | Annealing temperature (°C) | Application |
| --- | --- | --- | --- |
| mNOD1-F | GCAGAAGCTCCAGAACCTCTG | 52  52 | RT-PCR  RT-PCR |
| mNOD1-R  mNOD2-F  mNOD2-R | ATACTGTTGCCCCACATCCCA  TTGGATGACATTTACACTGATGG  ACAGGACCGATGTTACAGTAGGT |  |  |
| mNOD1 5' Router | CATCCTACAGCGGAACTT | 60 | 5' RACE-PCR |
| mNOD1 5' Rinner | AAGAAGAAGATGGTGTCCG |  |  |
| mNOD1 3' Router  mNOD1 3' Rinner | GTTGTGGAGGTCTTGGGGCTTTAC  GCTGCGAATGGTCAAGATCGGTAAA | 60  60 | 3' RACE-PCR  5' RACE-PCR |
| mNOD2 5' Router | CCGAGCCCAAAGCAAG |  |  |
| mNOD2 5' Rinner | GCTTCTGGAGTAAGGTGC |  |  |
| mNOD2 3' Router | GGGTCAGACAGGTGGTGAGATGCC | 60 | 3' RACE-PCR |
| mNOD2 3' Rinner | TCAACCTGTAGAGGGGGAGAAGAAG | 60 | qRT-PCR |
| qNOD1-F | GGGCTTTACAACAATCACATCA |  |  |
| qNOD1-R | GCCAAATACCTCCCACCAAC | 60 | qRT-PCR |
| qNOD2-F | GTCCTCTGCTGGATTGTCTCA |  |  |
| qNOD2-R | AGCCCCACAGTGCTCTTCA |  |  |
| β-actin-F | AGAGGGAAATCGTGCGTGAC | 60 | qRT-PCR |
| β-actin-R | ATACCGAGGAAGGAAGGCTG |  |  |
